# Fighting Epilepsy with Nanomedicines—Is This the Right Weapon?

**DOI:** 10.3390/pharmaceutics15020306

**Published:** 2023-01-17

**Authors:** Mariana Matias, Adriana O. Santos, Samuel Silvestre, Gilberto Alves

**Affiliations:** 1 CICS-UBI—Health Sciences Research Centre, University of Beira Interior, Av. Infante D. Henrique, 6200-506 Covilhã, Portugal; 2 CNC—Centre for Neuroscience and Cell Biology, University of Coimbra, 3004-517 Coimbra, Portugal

**Keywords:** antiseizure drugs, epilepsy, lipid-based nanosystems, polymeric nanoparticles, nanomedicines, nanotechnology

## Abstract

Epilepsy is a chronic and complex condition and is one of the most common neurological diseases, affecting about 50 million people worldwide. Pharmacological therapy has been, and is likely to remain, the main treatment approach for this disease. Although a large number of new antiseizure drugs (ASDs) has been introduced into the market in the last few years, many patients suffer from uncontrolled seizures, demanding the development of more effective therapies. Nanomedicines have emerged as a promising approach to deliver drugs to the brain, potentiating their therapeutic index. Moreover, nanomedicine has applied the knowledge of nanoscience, not only in disease treatment but also in prevention and diagnosis. In the current review, the general features and therapeutic management of epilepsy will be addressed, as well as the main barriers to overcome to obtain better antiseizure therapies. Furthermore, the role of nanomedicines as a valuable tool to selectively deliver drugs will be discussed, considering the ability of nanocarriers to deal with the less favourable physical-chemical properties of some ASDs, enhance their brain penetration, reduce the adverse effects, and circumvent the concerning drug resistance.

## 1. Introduction

According to the World Health Organisation, epilepsy affects around 50 million people worldwide, being a common, chronic, and serious neurological disease, strongly impacting patients’ quality of life [1,2]. Throughout the years, this pathology has held different definitions and epileptic seizures have undergone multiple updates in their classification [3,4,5]. Nowadays, epilepsy is defined by the International League Against Epilepsy (ILAE) as “a disorder of the brain characterized by an enduring predisposition to generate epileptic seizures, and by the neurobiologic, cognitive, psychological, and social consequences of this condition”. Epileptic seizures consist of the transient occurrence of signs and/or symptoms provoked by abnormally excessive or synchronous neuronal activity in the brain [5]. With advances in technology, genetics, and other fields, the term epilepsy is applied to an enormous variety of conditions and is very heterogeneous in its aetiology, clinical expression, severity, and prognosis. In fact, the clinical expression of epilepsy may include cognitive, behavioural, motor, autonomic, and other systemic impairments and dysfunctions [6,7]. Therefore, the complexity of epilepsy seems undisputed, and authors agree that its treatment remains an enormous challenge [8,9].

The process that involves the development and extension of brain tissue able to generate spontaneous seizures, leading to the development of an epileptic condition and/or progression for epilepsy, is known as epileptogenesis [9]. Several pathophysiological mechanisms have been associated with epileptogenesis, such as the transitory imbalance between the main neurotransmitters related with excitatory (glutamate) and inhibitory (γ-aminobutyric acid (GABA)) stimulus, as well as neuromodulators (e.g., dopamine, acetylcholine, norepinephrine, and serotonin) or alterations in type, number, or distribution of ion channels [10,11,12].

Epileptic seizures are unpredictable and can lead to damage, hospitalization, and, ultimately, death. An alarming fact is that epileptic patients have a mortality rate 2–3-times higher than the general population [13,14]. Moreover, seizures can also result in stigmatization, discrimination, and social exclusion, as well as an increased probability for the development of psychiatric comorbidities [14,15,16]. All these factors clearly support the need to find new strategies to effectively treat epilepsy, achieving a state of complete seizure freedom. Thereby, this review firstly focuses on the current therapeutic management of epilepsy and the hurdles found in the process of developing new drugs. Furthermore, the nanomedicines approach will be addressed as a new hope to develop safer and more efficacious antiseizure therapies.

## 2. Therapeutic Approaches

The main objective of epilepsy treatment is to achieve seizure freedom. In this context, long-term antiseizure drugs (ASDs) are, undoubtedly, the most used therapeutic approach, eliminating or reducing seizure frequency in around 70% of patients [17,18]. Currently, almost thirty ASDs are available in clinical practice, with high structural variety, different mechanisms of action and different physicochemical and pharmacokinetic properties, efficacy, and safety profiles [18,19,20]. Since the exact mechanisms underlying epilepsy are not well known, the drug treatment remains mainly symptomatic. In fact, available drugs are effective in stopping seizures (i.e., ASDs) but they are not curative and cannot stop the progression of epilepsy (i.e., they are not truly antiepileptic or antiepileptogenic drugs) [21]. Thus, although the terms “antiseizure drug” and “antiepileptic drug” are usually used as synonyms, this is not entirely correct. Indeed, “antiseizure” is a more accurate designation for these drugs [22]. It is the reason why it will be used throughout this review.

The selection of the most suitable ASD is challenging and several parameters need to be taken into consideration, such as the type(s) of seizure(s) or syndrome(s), the pharmacological properties of the ASD(s), and the individual features of the patient [23,24]. Monotherapy is the gold-standard strategy in epilepsy treatment, since the use of a single drug facilitates the efficacy evaluation, reduces the toxicity, avoids the risk of drug interactions, improves compliance, minimizes the costs, and allows the control of seizures in the majority of responsive patients [25,26]. However, in the cases of ineffective control of seizures with monotherapy, polytherapy (adjunctive therapy) should be considered, being as “rational” as possible. This concept includes the fact that ASDs with different mechanisms of action can act in a synergistic manner, providing better seizure control and reducing the potential of side effects compared to two drugs acting through a similar mechanism of action [7,27,28].

**Figure 1:**
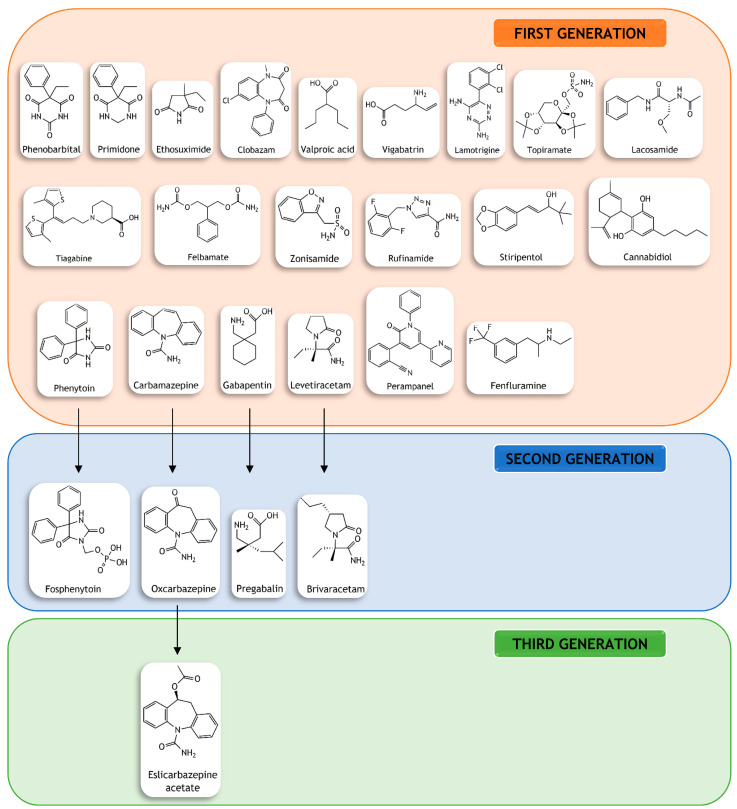
Structural diversity in chemical structures of the main clinically available antiseizure drugs. They were distributed into three consecutive generations according to their chemical structure and consecutive structural modifications to obtain drugs with improved properties [29,30].

The main ASDs that are currently available in clinical practice are illustrated in Figure 1, emphasizing the diversity in chemical structures. The distribution of ASDs into three consecutive generations was inspired by that described by Perucca et al. [29], which was based on the chemical advances and structural modifications of the already existing ASDs to produce new and more promising agents.

However, patients with drug-resistant epilepsy should be evaluated for non-pharmacological treatment options, such as surgery or specific dietary regimens [31,32,33,34].

The general goal of epilepsy surgery is the safe removal of epileptogenic brain tissues, which are those responsible for initiating seizures and whose resection or disconnection is essential for complete abolition of seizures [32,35]. When patients with drug-resistant epilepsy are not eligible for resective surgery, an alternative option is neurostimulation (e.g., vagal nerve stimulation and deep brain stimulation) [31,36]. Surgery is considered a highly effective therapeutic option for epilepsy, substantially improving cognition, behaviour, seizure control, and quality of life for patients. However, no epilepsy surgery should be carried out without preceding an in-depth evaluation [32,35,37,38]. Moreover, it has been described that after patients undergo the surgical procedure, about half of them require subsequent pharmacotherapy [32,35]. 

The ketogenic diet is a dietary regimen rich in lipids, with an adequate amount of proteins and low content of carbohydrates. It is a rigid and individually calculated diet, strictly monitored in order to produce ketone bodies in vivo [31,39]. Following the good results obtained with a ketogenic diet, other more specific dietary options have been considered for epilepsy, such as the medium-chain triglyceride ketogenic diet, the modified Atkin’s diet, and low-glycaemic-index treatment. These dietary regimens differ in the proportion of lipids, proteins, and carbohydrates, but have demonstrated similar efficacy to that observed with the classic ketogenic diet [40,41].

## 3. Barriers to the Development of New ASDs

Despite the large therapeutic arsenal of old and new ASDs, a high proportion of epileptic patients develops drug resistance during the course of the condition. Given the current status, there is a need for a more effective therapy for epilepsy. Therefore, it is essential to reverse the lack of interest that has been demonstrated by the pharmaceutical industry regarding the discovery and development of new therapies towards epilepsy [42]. Hence, several hurdles that need to be overcome to obtain better antiseizure therapies will be highlighted and discussed below.

### 3.1. Drug Resistance

The ILAE proposed, in 2010, a global consensus definition of drug-resistant epilepsy as “the failure of adequate trials of two tolerated, appropriately chosen and used AED schedules (either as monotherapies or in combination) to achieve sustained seizure freedom” [43]. This definition is based on the fact that if seizure control is not achieved with trials of two appropriate drugs, the likelihood of success with subsequent ASDs is much lower [7,43,44].

Drug resistance in epilepsy is associated with increased mortality, morbidity, reduced quality of life, and major financial implications [7,45]. When possible, epilepsy surgery, neuromodulation, and a ketogenic diet are alternative treatments for patients with drug-resistant epilepsy [32,44,46]. Moreover, broad-spectrum drugs with multiple mechanisms of action are potentially useful in some cases of drug-resistant epilepsy [28]. However, an important issue to take into consideration is that some patients do not adequately respond to ASDs for reasons other than drug resistance. They include, for example, the lack of compliance to pharmacotherapy and possible drug interactions that significantly affects ASD activity. Moreover, after the failure of monotherapies, inappropriate combinations of ASDs may also generate false-positive drug resistance profiles [47].

### 3.2. Loss of Efficacy

The modern era of ASDs, which started in the early 1990s, represented a new hope for achieving complete seizure freedom in a considerable number of patients who were drug resistant to older/classic drugs. Unfortunately, these expectations were not completely fulfilled and the overall probability of reaching a seizure-freedom state in the 21st century, with around 30 ASDs available in clinical practice, is approximately 70%. This value is similar to that observed in the early 1970s, when physicians had only half a dozen ASDs to prescribe [48]. Indeed, the introduction of new ASDs did not bring the expected incremental value to the pharmacological armamentarium; although most newer ASDs present a reduced toxicity burden and less potential for clinically significant drug–drug interactions, they are not more efficacious than older agents [49,50]. Some reasons have been proposed, such as the problem of drug resistance, the currently used animal models for the screening of new ASD candidates, and issues associated with clinical trial design [42,51,52,53,54,55,56,57].

### 3.3. Poor Safety Profile

Despite the advances in terms of tolerability and safety of recent ASDs, the pharmacologic treatment of epilepsy is frequently accompanied by long-term toxicity, dose-related severe side effects, and drug–drug interactions. These often result in a reduced quality of life, poor therapeutic adherence, or even discontinuation of therapy [21,58].

In terms of toxicity, central nervous system (CNS) adverse effects are a transversal problem in all ASDs. A possible explanation for this relates to the fact that these drugs were developed to target mechanisms of action that also interfere with normal neurotransmission [42]. In addition, the lower risk of hypersensitivity reactions and the lower potential for detrimental drug interactions may explain why some new ASDs (e.g., levetiracetam and gabapentin) are better tolerated than some classical ASDs (e.g., carbamazepine and phenytoin). However, serious idiosyncratic adverse effects have also been reported for several of the most recent drugs, such as vigabatrin (concentric visual-field defects) and felbamate (aplastic anaemia and hepatic failure), restricting their use [59,60]. In addition, a teratogenic potential has been reported for a number of drugs used to control seizures (e.g., valproic acid) [59,60,61,62].

Pharmacokinetic drug–drug interactions should also be highlighted, since they can lead to toxicity or reductions in the therapeutic effect. For example, some ASDs, such as phenytoin, carbamazepine, oxcarbazepine, eslicarbazepine acetate, or phenobarbital, induce cytochrome P450 isoenzymes, which results in a reduction in the concentration of other co-administered drugs [63].

### 3.4. Loss of Industry Enthusiasm

As referred to, until now, no ASD has demonstrated a prominent higher efficacy compared to any other, in particular against several types of epileptic seizures and syndromes, and differences in the safety profile are not the main reason for enhancing pricing and reimbursement [29,42]. Moreover, although an indication in monotherapy can move a drug to the market earlier, the approval of an ASD as monotherapy requires its previous approval as an add-on therapy, which leads to a substantial time delay [42].

On the other hand, it is important to highlight that the clinical heterogeneity in epilepsy could represent an opportunity for the pharmaceutical industry. Many types of epilepsy syndromes are associated with drug resistance, fulfilling the criteria for an orphan condition. In addition, some epilepsy syndromes have not approved ASDs for their treatment and, thus, drug candidates demonstrating any degree of efficacy can have benefits in terms of regulatory approval [48].

## 4. Are Nanomedicines the Solution?

The blood–brain barrier (BBB) is an important biological barrier that limits the delivery and/or the transport of ASDs to the brain, due to restrictive permeability and active efflux of some therapeutic agents. In this context, it has been proposed that problems in epilepsy treatment can be due to an insufficient concentration of ASDs in the CNS, which could be circumvented by suitable drug delivery systems. Therefore, nanometric delivery systems, usually referred to as nanocarriers or, lato sensu, nanoparticles (NPs), aim to provide an increased drug biodistribution to the brain, thus, reducing the peripheral drug-associated toxicity and augmenting the drug’s effectiveness [64]. In addition, NPs can also solve solubility and bioavailability issues in therapeutic agents and protect them from enzymatic degradation [65].

NPs, according to their formulation, may display unique structural, physicochemical, magnetic, and electrical properties, making them unique tools for drug delivery. In general, the size, surface area, and charge, targeting ligands and the morphology of nanocarriers, can have a significant impact on their penetration of CNS barriers [66,67,68]. For example, it is described that NPs with a positive surface charge establish electrostatic interactions with the negative surface charge of endothelial cells in the brain. Moreover, the lipophilic nature of NPs also enhance and facilitate the adsorption process [68]. Receptor-mediated transcytosis in brain endothelial cells can be triggered by the adsorption of plasma apolipoproteins to mask certain NPs [69] or by the use of specific targeting ligands that are specific to receptors or transporters overexpressed in the BBB, such as glucose, transferrin, and angiopep-2 (ANG) [70]. Taking into account that nanocarriers are able to mask molecules, allowing for their passage to the cerebral parenchyma across the BBB, they have also been considered important tools for the treatment of drug-resistant diseases, namely brain tumours or other CNS disorders, such as Alzheimer’s and Parkinson’s diseases [71].

**Figure 2:**
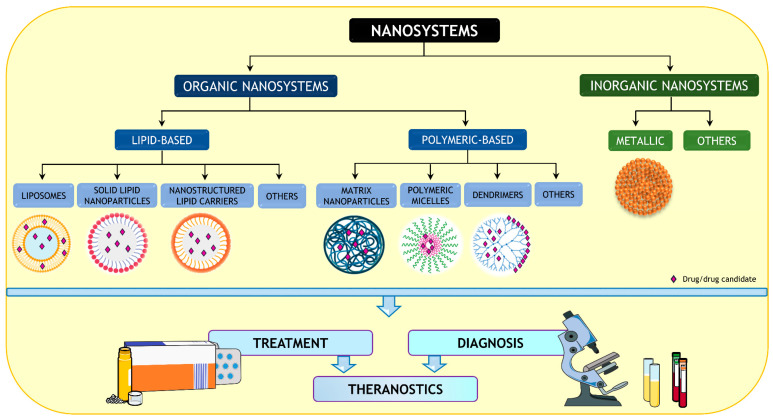
Overview of nanodelivery systems used to formulate antiseizure drugs. Part of the figure was drawn by using pictures from Servier Medical Art. Servier Medical Art by Servier is licensed under a Creative Commons Attribution 3.0 Unported License (https://creativecommons.org/licenses/by/3.0/ (accessed on 31 August 2022)).

Different drug delivery systems are available nowadays, such as polymeric NPs, liposomes, solid-lipid nanoparticles (SLNs), nanostructured lipid carriers (NLCs), metallic NPs, among others [65,68]. In Figure 2, a general overview of drug delivery systems is presented, focusing on the main types of NPs explored pre-clinically in the context of epilepsy treatment and diagnosis.

Polymeric NPs are solid colloidal dispersions of biocompatible and biodegradable polymers, which encapsulate and deliver diverse types of therapeutic agents, including large biological macromolecules, such as proteins. For the preparation of these NPs, either natural or synthetic polymers can be used [68]. Examples of natural polymers are chitosan (CH), gelatine, sodium alginate, and albumin. Regarding synthetic polymers, the most used are polylactic acid (PLA), polyglycolic acid (PGA), poly(lactic-co-glycolic acid) (PLGA), polyanhydrides, polycyanoacrylates, polycaprolactone (PCL), poly-*N*-vinyl pyrrolidone, and polyvinyl alcohol [66]. In the strictum sense, authors usually refer to the matrix type as simply “polymeric NPs”, consisting of a core of a dense insoluble polymer matrix. They could be added to a hydrophilic corona (coat) to provide steric stability. In addition, coating with polyethylene glycol (PEG) could prevent the rapid clearance from the systemic circulation by cells in the mononuclear phagocytic system, prolonging the NP circulating time [67]. Drugs can be encapsulated within the matrix, adsorbed, or chemically linked to the NP surface. Among them, PLGA is a good example of an FDA-approved polymer used to obtain such particles that has allowed for a controlled and sustained drug release at the target sites. Other types of NPs made of polymers exist, such as “Nanocapsules” (a polymer coating a drug reservoir) or the self-assembling polymersomes and polymeric micelles, made of amphiphilic polymers, usually block copolymers. Regarding dendrimers, they are radially symmetric branched polymers that have smaller hydrodynamic and molecular volume in comparison with linear polymers of similar molecular weight. These polymeric molecules have a well-defined, homogeneous, and monodisperse structure, which consists of tree-like arms or branches [72,73]. Several advantages have been attributed to this type of polymeric NP, such as prolonged circulation time, high aqueous solubility, biocompatibility, polyvalency, increased stability of the compound, and ability to transport a wide range of molecules, either for treatment or diagnosis [68,72].

Although polymeric NPs have been mainly explored for the delivery of drugs/drug candidates with antiseizure properties, several lipid-based nanosystems have also been investigated with interesting results. In general, classic lipid-based NPs are flexible, biocompatible, biodegradable, and the least dangerous NPs, and are, thus, suitable for in vivo applications. Indeed, liposomes are commonly made of phospholipids found in the organism, as well as cholesterol [66]. Liposomes are the best-known and most versatile vehicle among lipid-based nanocarriers. They are formed by one or more concentric lipid bilayers separated by aqueous compartments [74]. The molecules may be encapsulated into the aqueous centre (hydrophilic compounds) or incorporated into the bilayer membrane in the case of lipid-soluble agents. The efficacy of therapeutic drugs loaded in liposomes is demonstrated by several liposomal formulations in clinical use [74,75]. Although liposomes are the most popular lipid-based nanosystem, they often suffer from reduced drug loading capacity, short half-life, enzymatic degradation, and poor stability. Thus, SLNs and NLCs emerged as liposome substitutes to counteract these limitations. SLNs are aqueous colloidal nanocarriers formed by natural lipids, such as triglycerides, fatty acids, and steroids, dispersed in an aqueous solution [65,68]. Both hydrophilic and hydrophobic agents can be dispersed in the solid hydrophobic lipid core [76]. They have been considered safer than polymeric NPs due to the absence of organic solvents during their production and have demonstrated a more prolonged drug release and higher stability than liposomes [65]. On the other hand, some limitations, such as uncertain gelation tendency, uneven or inadequate drug incorporation, and expulsion of incorporated agents led to the production of NLCs. These nanocarriers are aqueous dispersions of NPs, with a solid lipid matrix constituted by one solid lipid, one liquid lipid, and stabilizing emulsifiers. The oil within the lipid matrix leads to an imperfect structure, increasing the stability [77]. The blend of liquid and solid lipids facilitates the encapsulation of hydrophobic agents due to improved drugs solubility. However, the loading of hydrophilic drugs into NLCs and the encapsulation of two or more therapeutic molecules have been a challenge [65].

Metallic NPs have also been explored in the epilepsy field. Indeed, they present several advantages, such as their small size-to-volume ratio, dense surface functionalization, stabilization, and ease of detection, which make them useful not only for treatment but also for diagnosis [78]. Different metals have been used in the preparation of these NPs, such as silver, gold, aluminium, copper, iron, and zinc. Due to the fact that there are some concerns for human health due to the environmental problems regarding some metals, the concept of green metallic NPs has emerged and efforts have been made to produce metallic NPs with good safety profiles and low environmental impact [79,80]. Moreover, a highlight also goes to theragnostics, which are systems, including both applications of treatment and diagnosis [81]. An example is given by Long et al., as described later in this manuscript [82].

Another type of NP, although less explored in the context of epilepsy, is drug nanocrystals. However, it is noteworthy to mention this strategy of formulation since it allows for the modification of the physicochemical properties of the drug/drug candidates, leading to important effects, such as enhancements in bioavailability. These NPs are commonly used as a synonym of “nanosuspension” and basically consist of nanosized particles of the compounds, with crystalline characteristics [83].

Overall, in the next sections, the advantages of using different nanocarriers to overcome some limitations of currently available ASDs or ASD candidates (those not approved for epilepsy by regulatory agencies) are described and critically discussed. Even if some advantages can co-exist, the examples are discussed by a particular achievement, either an increase in brain penetration or higher brain bioavailability, increased formulation drug strength by solving low aqueous solubility limitations, reduction in drug-associated side effects, and counteraction of brain mechanisms of drug resistance. An important amount of papers described the advantages of nanosystem use, such as an improvement in aqueous drug strength/physicochemical properties, decrease in administration frequency, and reduction in ASD side effects. However, the truth is that a relevant number of the studies found in the literature did not investigate whether the researched nanocarrier was, indeed, an effective tool to solve these problems in that particular case. These papers were excluded from the results of this review as well as those that did not compare the formulated compound in study with its unformulated form as a control.

### 4.1. Nanoformulated Antiseizure Drugs

#### 4.1.1. Increase in Drug Brain Penetration

Most studies, including ASDs loaded in NPs, refer to the increase in brain penetration as the main goal. These findings are often demonstrated through the determination of pharmacokinetic parameters of the drug-loaded NP in comparison with those of its free form. In this context, Ammar et al. prepared lamotrigine-loaded poly-ε-(d,l-lactic-co-caprolactone) NPs through the spontaneous emulsification solvent diffusion method. The NPs presented a size of 125 nm, considered by the authors as sufficiently low to promote BBB penetration, without being taken by the reticule-endothelial system. Pharmacokinetic studies showed that intravenous (i.v.) lamotrigine NPs exhibited higher values of maximum time and concentration, area under the curve, and mean residence time in homogenized rat brain, compared to the oral unformulated drug. Although lamotrigine’s NP bioavailability in rat plasma was lower than oral lamotrigine, the results of this experiment confirmed that the NPs used were an effective system for lamotrigine brain delivery (entry 1, Table 1) [84].

Another ASD, phenytoin sodium, was formulated into electro-responsive hydrogel NPs (ERHNPs), made of the monomers 2-dimethylamino ethyl methacrylate, sodium 4-vinylbenzene sulfonate, styrene, and acrylate-poly(ethylene glycol)-*N*-hydroxysuccinimidylester and *N*,*N*′-methylene bisacrylamide as the cross linker and modified with ANG to enhance its delivery, as proved by in vitro and in vivo assays. Regarding the studies using an animal model, high concentrations of phenytoin were found in regions of the brain associated with seizure initiation and spread, such as hippocampus, amygdale, cerebellum, and brainstem. Moreover, 15 min after treatment with the NPs, without or with ANG, the phenytoin levels in the brain were 1.49- and 1.97-fold higher, respectively, compared with unformulated phenytoin. Importantly, the authors associated these results to the ANG brain targeting effects, to the NP small dimensions (~130 nm), and to the prolonged blood circulation time promoted by PEG. In addition, the phenytoin-loaded ANG-ERHNPs displayed antiseizure effects in an amygdala kindling model of epilepsy (entry 2, Table 1), which also proved that these NPs were able to transport ASDs into the brain [85]. In order to clarify the effect of particles’ electroresponsive ability in antiseizure activity, the same research group developed nonelectroresponsive hydrogel NPs (ANG-HNPs). Although they showed protection against electric- and chemical-induced seizures, phenytoin included into ANG-ERHNPs displayed improved antiseizure properties compared to ANG-HNPs and the unformulated form. Pharmacokinetic studies demonstrated that free phenytoin concentration in brain dialysate 30 min after pentylenetetrazole (PTZ)-induced seizures was significantly higher in the ANG-ERHNPs compared with ANG-HNPs and free phenytoin groups. This indicated that phenytoin release from ANG-ERHNPs was triggered by seizures [96]. Gabapentin was formulated into albumin NPs, aiming to improve its effectiveness towards epilepsy. Polysorbate-80-coated NPs increased brain’s gabapentin concentration around 3-fold, compared to free ASD, which probably was associated with their higher antiseizure activity against electric- and chemical-induced seizures than both the unformulated drug and gabapentin-loaded albumin particles without polysorbate 80 (entry 3, Table 1) [86].

The incorporation of carbamazepine in SLNs (entry 4, Table 1) demonstrated seizure protection after oral administration both against acute and chronic animal models. A further histopathological analysis of mice hippocampus showed a reduction in the percentage of degenerative cells by carbamazepine SLNs when compared with free drug. In addition, a prolonged antiseizure activity of the nanoformulation was associated with sustained drug release, which was demonstrated in vitro, and to an improvement in the absorption profile and penetration of the nanosized particles. However, this last suggestion was not experimentally demonstrated [87].

The ASD stiripentol was loaded into nanosuspensions stabilized with denatured soybean protein isolate, with a mean size of 150 nm. This new formulation was able to permeate across the Caco-2 cell monolayer as well as to cross the adherent mucus layer and penetrate enterocytes. These results were confirmed by the good oral bioavailability and fast absorption in nanosuspensions of stiripentol in rats (entry 5, Table 1) [88].

Metallic NPs were also investigated in the context of epilepsy treatment. An example is given by Yilmaz et al., who developed glucose-coated gold NPs to incorporate lacosamide, aiming to potentiate its brain penetration (entry 6, Table 1). The results showed that higher levels of NPs were found in the hippocampus of epileptic rats when compared with healthy animals. Regarding efficacy, the authors reported that lacosamide-loaded NPs decreased the amplitude and frequency of electroencephalography waves in both ictal and interictal stages in the rats with temporal lobe epilepsy [89].

#### 4.1.2. Reduction in Drug Adverse Effects

A nanosystem based on negatively charged dendrimers was developed to deliver carbamazepine (entry 7, Table 1). It was found that 4.5 PAMAM dendrimers (DG4.5), incorporating carbamazepine into their hydrophobic pocket, demonstrated no haemolytic effect nor morphological changes in human red blood cells in an ex vivo model and lower toxicity in vitro in N2a cells compared with the free drug. Additionally, the DG4.5-carbamazepine did not cause neurotoxicity or cardiotoxicity (e.g., alterations in heart rate) in zebrafish larvae [90].

#### 4.1.3. Overcoming P-gp-Mediated Drug Resistance

An interesting study was carried out by Fang and co-workers, who produced polymeric NPs bearing phenytoin, aiming to overcome the concerning drug resistance. The poloxamer, with the commercial name Pluronic^®^ P85, was used to coat phenytoin poly(-butylcyanoacrylate) NPs, which showed higher antiseizure activity in phenytoin-resistant rats than the free drug and similar efficacy to the combination of phenytoin and tariquidar, a P-gp inhibitor. Further, nanoformulated phenytoin led to a higher drug distribution (2-fold) into the brain, compared to unformulated drug (entry 8, Table 1) [91]. This ASD was also formulated in a silica core of iron oxide NPs and evaluated using rats that received repetitive administration of 3-mercaptopropionic acid to induce P-gp overexpression. An increase was found in the antiseizure effect of phenytoin-loaded NPs in this animal model of drug resistant epilepsy, compared with the free drug (entry 9, Table 1) [92]. These findings were of particular importance, since they suggested that the nanocarrier loading phenytoin was able to circumvent resistance to phenytoin in epileptic rats with P-gp overexpression.

Furthermore, Zybina et al., in 2018, investigated whether verapamil, a P-gp inhibitor, interfered with the antiseizure activity of carbamazepine and its nanoparticulate formulation by inhibiting P-gp. For this, a rat model of isoniazid-induced epilepsy was used. The antiseizure effect of carbamazepine was increased after the administration of verapamil, which suggested that this ASD is a P-gp substrate. Due to the P-gp inhibition, its effective dose was reduced by at least 30% (from 30 mg/kg to 20 mg/kg). In an attempt to overcome drug resistance promoted by P-gp overexpression, carbamazepine was loaded into poloxamer-188-coated PLGA NPs. The results of this study showed a 30-fold increase in antiseizure properties, compared to unformulated carbamazepine (entry 10, Table 1). For this reason, authors suggested that the efficacy of carbamazepine encapsulated in NPs was not influenced by P-gp, which can represent a strategy to overcome drug resistance [93]. An interesting result from this investigation was the effect of carbamazepine-loaded NPs without the surfactant poloxamer 188 that was similar to placebo, demonstrating the important role of this surfactant for brain delivery considering this formulation. An additional experiment showed an increase in the in vitro penetration of carbamazepine incorporated in SLNs and NLCs using P-gp-expressing cells. However, the assay also demonstrated that carbamazepine NLCs only slightly protected a little more than the unformulated drug [97].

Liu and collaborators incorporated lamotrigine in mixed polymeric micelles made of two poloxamers (Pluronic^®^ P123/F127) and functionalized with a tryptophan derivate, aiming to successfully deliver the ASD to epileptogenic focus, while overcoming multidrug resistance by P-gp modulation. The small NPs (<30 nm) were intravenously administered in a rat model of *status epilepticus* induced by pilocarpine and the brain uptake efficiency of the lamotrigine loaded in polymeric micelles was measured (entry 11, Table 1). The results showed that this nanoformulation was more efficient in delivering the ASD to the hippocampus than the free ASD. Moreover, the authors suggested that the ingredients used in the formulation increased the cellular uptake of the P-gp substrate rhodamine 123, demonstrating their role in the inhibition of this efflux transporter [94]. Another relevant finding in this study was the demonstration of P-gp overexpression in the chronic epilepsy model used. This finding, together with the experimental results of higher brain penetration of lamotrigine-loaded micelles than the free drug, reinforces the ability of the formulation to be effective in overcoming drug resistance associated with the overexpression of P-gp.

### 4.2. Nanoformulated Antiseizure Drug Candidates

#### 4.2.1. Increase in Compound’s Brain Penetration

Several authors also investigated the delivery and efficacy of particularly problematic natural compounds not yet approved for anticonvulsive therapy (Table 2). Epigallocatechin-3-gallate (EGCG), a natural polyphenol known for its rapid degradation, was loaded in PEGylated-PLGA NPs via the double-emulsion method, aiming to protect it and to increase its brain delivery. Interestingly, NPs presented 95% encapsulation efficiency and a sustained release profile. Opposed to free compound, the differential scanning calorimetry showed the absence of peaks corresponding to the process of drug melting decomposition on formulated EGCG. This result suggested that the compound incorporation in the polymeric NPs in the study effectively protected the compound from degradation. In addition, pharmacokinetic studies demonstrated that the free compound displayed a faster absorption than encapsulated EGCG. However, the NP containing EGCG maintained plasma levels up to 240 min after its administration, while the unformulated compound was undetectable after 90 min. These results also suggested protection against early degradation of the drug candidate. The EGCG NPs also reduced the number and severity of seizures to a higher extent than the free compound (entry 1, Table 2). Moreover, a reduction in neuroinflammation and neuronal death was observed [98].

Another natural product, piperine, which can be isolated from *Piper longum* L., has demonstrated antiseizure and neuroprotective activity [104]. Through the nanoprecipitation method, piperine NPs, made of a pH-sensitive methacrylic acid polymer (Eudragit S100) and poloxamer 188, were produced with an entrapment efficiency of 92.2 ± 2.5%. Oral piperine NPs, described in entry 2, Table 2, displayed higher protection towards PTZ-induced acute seizures in both zebrafish and mice models than the unformulated compound. In addition, pharmacokinetic studies demonstrated that after oral administration, piperine NPs improved (2.7-fold) the oral bioavailability and led to higher concentrations (i.e., 16-times) of this bioactive compound in the brain at 10 h post-dosing [99]. Curcumin, another natural product, was loaded in SLNs (entry 3, Table 2) and this nanoformulation in vitro decreased the level of free radicals and reversed mitochondrial function through the activation of the Bcl-2 family, protecting against neuronal apoptosis to a higher extent than unformulated curcumin. Further, in vivo studies demonstrated that SLNs improved the penetration of curcumin through the BBB, as proved by the higher concentration of the natural compound when administered in its nanoformulated form [100]. Although this study did not show the antiseizure effect of curcumin, other studies, such as that reported in Table 2, entry 5, demonstrated that this natural product protects against tonic–clonic seizures.

Taking into account the important role of GABA in epilepsy genesis and its limitation crossing the BBB due to its high hydrophilicity [105], its formulation into *N*,*N*-dimethylacrylamide-based PEGylated NPs was proposed aiming its delivery into the brain. Using an acute seizure model, the nanoformulated GABA displayed better results than the unformulated neurotransmitter, as shown in entry 4, Table 2. Curiously, GABA concentration measured in *Stratum corsatum* was similar for both formulations [101]. However, the difference in seizure protection between the two groups of rats could be explained by the delivery of GABA in other regions of the brain related to seizure focus. GABA was also loaded in liposomes composed of phosphatidylserine. This formulation displayed antiseizure activity, contrarily to free GABA that did not produce any significant effects, using an isoniazid-induced seizure model [106]. Although the authors attributed these results to a higher brain penetration for liposomal GABA, they did not perform any experiment to prove this hypothesis.

#### 4.2.2. Overcoming Compound’s Poor Water Solubility

The drug’s poor aqueous solubility is often the limiting step for drug absorption at oral administration, hence, hampering the pharmacological effects of bioactive compounds [107]. For this reason, the development of strategies aiming to improve the (apparent) water solubility of known drugs or other compounds with high therapeutic potential is crucial. In this context, Hashemian et al. loaded curcumin in CH–alginate–sodium tripolyphosphate NPs. Indeed, this strategy significantly increased formulated curcumin content, compared with the unformulated compound (520.3 ± 96.52 μg/mL versus 10.94 ± 0.38 μg/mL, respectively). In addition, the results showed that NPs bearing curcumin exhibited higher seizure protection in a mice model of chronic epilepsy (entry 8, Table 2) in comparison with the unformulated compound. Moreover, a reduction in the level of cell death and glial activation was found in animals receiving nanoformulated curcumin [102]. Furthermore, in an attempt to elucidate the mechanism of action of curcumin-loaded NPs, the authors showed that they were able to downregulate the tumour necrosis factor alpha (TNF-α) and upregulate the levels of klotho, a life-extension factor, and erythropoietin in the same animal model of epilepsy [108].

#### 4.2.3. Reduction in Compound’s Toxicity

A lipid-based nanosystem, designed as lipid–protein–sugar particles (LPSPs) containing muscimol, was investigated regarding its potential for epilepsy control [103]. Muscimol is a GABA_A_ receptor agonist with reported antiseizure activity, including against drug-resistant epilepsy [109,110]. Muscinol, in both its nanoformulated and free forms, was evaluated in a rat model of intrahippocampal pilocarpine after injection into animals’ hippocampus. Ex vivo studies showed a reduced neuronal injury with significantly less apparent cell loss for encapsulated muscimol, compared with the free compound. However, no significant difference in protective effects was found between the formulations (entry 9, Table 2) [103]. Although the authors did not emphasise this finding, we highlight the fact that in the animals belonging to the unencapsulated groups, the mortality was considerably higher, compared to the nanoformulated compound. For this reason, we suggest that, in this case, the lipid nanocarrier used was effective in reducing the toxicity of the tested compound.

### 4.3. Intranasal Administration

Several studies considering the intranasal (i.n.) administration of drugs/drug candidates, either nanoformulated or in their free form, are available in the literature (Table 3). Indeed, the i.n. route has been explored in the recent years as a non-invasive alternative to deliver therapeutic agents to the brain, because compounds applied in the nasal cavity can directly reach the brain through the olfactory mucosa. In this context, Musumeci et al. administered oxcarbazepine via the i.n. route and confirmed its antiseizure properties at 0.5 mg/kg (1 dose, every 20 min for 1 h) in a PTZ model of seizures. Furthermore, oxcarbazepine was loaded into PLGA NPs aiming to reduce the number of daily administrations. This formulation allowed for a reduction in the number of administrations to one over 24 h, when compared with the free drug, without loss of the antiseizure activity (entry 1, Table 3). Moreover, the neuroprotective effect of this nanoformulation was confirmed by immunohistochemical assays after 16 days of treatment [111]. An additional study used carboxymethyl CH NPs as a carrier to deliver carbamazepine via the i.n. route (entry 2, Table 3). The results showed that the i.n. administration of carbamazepine-loaded NPs in mice markedly increased the concentrations of this ASD in plasma and brain, in comparison with the concentrations found after the administration of free carbamazepine. These results suggested that the drug uptake from nasal mucosa was by the olfactory pathway, since carbamazepine concentrations were higher in the brain compared to plasma. In addition, the uptake was also achieved from the systemic pathway, where the drug enters into the systemic circulation and reaches the brain through the penetration of the BBB [112]. Clonazepam was also loaded in both lipid-based nanocarriers and administered via the i.n. route after incorporation in a thermosensitive mucoadhesive in situ gel. The NLC formulation containing clonazepam demonstrated good antiseizure activity (Table 3, entry 3). Furthermore, it was loaded in superparamagnetic iron oxide NPs, which protected the animals in the study against chemical-induced seizures to a higher extent. These results proved that, under an external magnetic field, drug carriers with enclosed magnetic NPs can be guided towards targeted tissues, enhancing the therapeutic effects [113]. A different approach was followed by Pires et al., who combined the insoluble phenytoin and the respective soluble prodrug (fosphenytoin) in a nanometric emulsion containing albumin and high drug strength (34.6 mg/g of phenytoin equivalents) (Table 3, entry 4). This approach resulted in a high absolute blood bioavailability (141%), mainly due to higher drug levels at longer time points (from 4 h to 8 h). In addition, the nanoformulation resulted in a much faster absorption compared to the free prodrug in solution, reaching, at 15 min, an equivalent brain concentration to that obtained with i.n. free fosphenytoin only at 60 min post-administration [114].

Regarding non-approved compounds for epilepsy, a nanoemulsion, with the composition described in Table 3, entry 5, was prepared incorporating the drug amiloride. This nanoformulation showed higher protection against both electrical- and chemical-induced seizures after i.n. administration in mice. In addition, the amiloride-loaded nanoemulsion demonstrated a higher brain concentration in comparison with i.v. administration, as well as higher area under the curve in the brain, lungs, and plasma when compared to the unformulated drug [115]. Amiloride is a potassium-sparing diuretic and its mechanism of antiseizure activity remains to be elucidated. However, it has been described that the compound can act through the inhibition of voltage-gated sodium channels or the blockade of the acid-sensing ion channels ASIC1a and ASIC3, especially ASIC1a [118,119].

Two additional studies, considering the i.n. route, were carried out by the same research team, aiming to formulate two natural products in polymeric NPs in order to improve their bioavailability. In the first, these authors developed CH-coated PLGA NPs to deliver catechin hydrate. The NPs were prepared through a double-emulsion-solvent evaporation method, followed by coating with CH and obtained with an entrapment efficiency of around 74%. The NPs containing catechin exhibited better permeation via nasal mucosa compared with the NPs without CH and with the free compound (permeation of > 83.64 ± 5.33, 68.64 ± 4.66 and 20.34 ± 2.99%, respectively) and good mucoadhesive nature. These NPs also showed significant protection either in electrically or chemically induced seizures in rats (entry 6, Table 3) [116]. The same method was used to produce PLGA NPs bearing thymoquinone, the main bioactive constituent in *Nigella sativa*. The i.n. administration of the formulation showed an improved brain bioavailability of thymoquinone when compared to i.n. administration of this compound in its free form (98.24 ± 6.89 versus 6.55 ± 0.83%, respectively) and to the i.v. route. Moreover, it also displayed an enhanced seizure threshold in an increasing current electroshock rat model (entry 7, Table 3) [117]. In this context, it has been reported that the antiseizure mechanism of action of thymoquinone can be related to an opioid-receptor-mediated increase in GABAergic tone [120].

## 5. Nanoparticles for Diagnosis and Theragnostics

Beyond treatment, NPs have also been studied for diagnosis in the epilepsy field, as already mentioned. These can be particularly useful for drug resistant epileptic patients that are elected for surgery. Indeed, one of the criteria used to determine eligibility for resective epilepsy surgery is the epileptogenic zone being well localised [36]. In addition, the surrogate marker should also predict which patients will probably develop epilepsy after a potentially epileptogenic brain injury and determine which therapeutic option is likely to be effective in each patient [121].

Considering these objectives, a nonradioactive alpha methyl tryptophan (AMT) was covalently attached to magnetic NPs constituted by iron oxide and dextran, in order to accumulate in epileptic regions of rats, making them visible through magnetic resonance imaging (MRI). These NPs were injected into the tail vein of Lewis rats of a model of temporal lobe epilepsy induced by kainic acid. As shown by MRI, the AMT NPs crossed the BBB, located in both hippocampi in the acute seizure model. An important finding of this study was the fact that AMT NPs accumulated in the brains of rats exhibiting spontaneous seizures (chronic epilepsy model), but not in the rats without spontaneous seizures (acute seizure model), demonstrating their potential to be a specific surrogate marker of epileptic regions of the brain [121]. Superparamagnetic NPs were also proposed by Pedram and collaborators for epileptic area detection. The authors suggested that after crossing the BBB through magnetic forces, NPs can aggregate and be used as a marker to increase the contrast in MRI images. This is more evident in epileptic areas, where the increased activity of neurons leads to increasing current flowing through, also increasing magnetic force, leading to NP aggregation [122]. In another study, PLA-based magnetite-impregnated NPs were prepared and injected intravenously to Wistar rats with lithium-pilocarpine-induced epilepsy (chronic epilepsy animal model). These NPs were fluorescently labelled, selectively accumulated within myeloid cells in hippocampal tissue, in regions of known seizure activity and neuronal loss in this animal model. Interestingly, the authors reported that these NPs concentrated in the hippocampus that is the seizure origin and not in the regions induced by the spread of seizure activity, such as thalamus and cortex [123].

Another magnetic-targeted drug delivery system with superparamagnetic iron oxide NPs and including an anti-interleukin-1β monoclonal antibody (anti-IL-1β mAb) was tested in an acute rat model of temporal lobe epilepsy. However, in this case, the objective was the diagnosis through imaging techniques (MRI) and the treatment with the anti-IL-1β mAb that has been proposed as an antiepileptogenic therapeutic agent. After i.v. administration in Sprague-Dawley rats, the NPs crossed the BBB and accumulated in the neurons and astrocytes in epileptogenic tissues. Moreover, the drug candidate led to an improvement in the organizational structure in the hippocampal CA3 area, which was altered by the administration of pilocarpine [124]. In addition, bone marrow mesenchymal stem cells pre-labelled with ultrasmall superparamagnetic iron oxide NPs were investigated in a rat model of temporal lobe epilepsy induced by lithium-pilocarpine using MRI. These NPs were found in the hippocampus and adjacent parahippocampal cortical areas of the rats and they did not alter the biological characteristics of the stem cells under study. Moreover, antiseizure activity was also exhibited by the nanoformulation, confirming its use in theragnostics [82]. These agents are able to reduce the timespan for the treatment of epileptic patients, monitoring the biodistribution of the NPs in the body [81].

## 6. Critical Overview

Taking advantage of the potential use of the discussed NPs, different limitations of the current clinical available ASDs could be overcome. For example, the premature carbamazepine metabolism could be prevented by its nanoformulation. This might be beneficial to reduce the conversion of this drug to its 10,11-epoxide metabolite that has been associated with important toxic effects, namely seizure exacerbation, *status epilepticus*, and hypersensitivity reactions [125,126,127]. In addition, the action of sodium valproate can also be limited due to its poor BBB penetration, side effects, and rapid tissue metabolism [128]. Theoretically, all these factors could be ameliorated by their nanoformulation in an appropriate NP. Beyond ASDs in clinical use (Table 1), as demonstrated in this review, the antiseizure effect of different natural products has also been investigated (Table 2). In these cases, the main limitations proposed by the authors are their problematic physicochemical properties that limit the molecules’ solubility in physiological fluids or their barrier crossing, reducing their bioavailability and, consequently, the desired biological effects. In this context, nanocarriers could serve as an important tool to improve the pharmacokinetic profile of these compounds that appears as the main barrier to their antiseizure action. Indeed, in general, improvements in the pharmacokinetic parameters translates to better pharmacodynamic effects. However, we proposed that a deeper investigation might be performed to understand the real advantages of nanomedicines and how these approaches lead to a better antiseizure profile of formulated compounds in comparison with the corresponding free form.

Particularly, information about the systemic toxicity, biocompatibility, accumulation, and excretion of NPs remains unclear and is difficult to generalize. It is clear that small changes in NP compositions can lead to dramatic alterations in their stability, compromising the desired therapeutic effects and security. Inorganic NPs are generally the most problematic (toxic). In contrast, biodegradable NPs made of biocompatible lipids and polymers can be naturally degraded into non-toxic bioproducts in the human body. Moreover, they can be designed for their degradation only at the target site, remaining stable at off-target regions [129]. For example, PLA can be hydrolysed into lactic acid under physiological conditions and PLGA into lactic and glycolic acids and, after, being degraded into non-toxic products, such as water and carbon dioxide, that are easily excreted (reviewed in [130]). Interestingly, adjusting the ratio of PLA:PGA and the molecular weights can change the degradation rate of PLGA and, consequently, control the release of the loaded molecules. The authors also reported that the biodistribution and pharmacokinetics of PLGA are easily affected by different factors, such as the hydrophilicity, the chemical interactions, the crystallinity, and the volume to surface ratio of PLGA. Moreover, CH has been shown to be very sensitive to temperature and pH, influencing its stability [130]. Thus, stability studies considering the degradation of NPs before reaching the targeted site are highly recommended, since it increases the risk of premature drug release to an off-site target, affecting the efficacy of the therapeutic agent.

Regarding lipid-based nanosystems, many examples, such as classic liposomes, have been considered non-toxic, since most of the phospholipids and cholesterol used in their composition are found in the human body, such as in cell membranes. However, not all lipid-based nanosystems are composed only of these types of lipids. Moreover, a biodegradable NP is not a synonym for non-toxic NP.

All of these factors make nanomedicines a complex matter and demand a more in-depth knowledge about the intrinsic toxicity of NPs, their accumulation in the human body, and elimination pathways. In addition to working with safer materials, since toxicity is usually dependent on the dose, the way out could be to use them only when the benefit outweighs the risk, make sure to improve drug loading, and optimize biodistribution/targeting in order to reduce the NP co-administered dose. The routes of drug administration used in the studies should also be highlighted. The oral administration is the most common route of delivering ASDs to patients, allowing for self-administration and enhancing the chances of good compliance. Thus, the design of drugs capable of being orally administered remains the focus of the majority of small-molecule drug discovery projects [131]. For this reason, it was surprising to observe that an important percentage of the studies found in the literature on this topic used the i.v. and i.p. routes of administration. Particularly, in the context of nanotechnology, this issue should be carefully addressed due to the suitable selection of the ingredients in the nanoformulation to deliver drugs via the desired route. Even the studies that evaluate the formulations through the i.v. route, which is indicated for *status epilepticus*, did not compare with the available drugs usually administered for this condition (e.g., benzodiazepines). On the other hand, the investigation of the i.n. route should be encouraged, since it is a non-invasive alternative, offering enhanced targeting and reduced toxicity. Indeed, if the drugs are less absorbed in the blood stream, the systemic exposure and the risk to produce undesired side effects is diminished [132]. In the context of drug resistance, the studies using NPs as a strategy to solve this problem were based on the role of P-gp and its overexpression in the BBB. We believe that an alternative to circumvent this important biological barrier, such as the i.n. route, can represent a way to improve this concerning ASD resistance. However, it is important not forget that the phenomenon of drug resistance is complex in its nature and undoubtedly incompletely known. Therefore, although the transporter hypothesis is the most popular, other theories have been associated with drug resistance, such as genetic or acquired alterations in the structure and/or functionality of cellular targets of ASDs; the increase in severity over the years reflects the magnitude of the underlying epileptic process and genetic variations [9,42,56,133,134,135,136].

Regarding the discovery and development of new ASD candidates and taking into account the impact of drug resistance, the Epilepsy Therapy Screening Program released by the National Institute of Neurological Disorders and Stroke (NINDS) now recommends a 6 Hz animal model to be used in the initial phases of identification of new drug candidates for epilepsy. Thus, it was surprising that there was no study using this model of seizures. For this reason, we emphasize the importance of considering the introduction of acute and/or chronic animal models of drug resistance (either chemically or electrically induced), since these models tend to better mimic the human condition of the disease based on the presence of responders and nonresponders. Indeed, since the progress in the molecular and genetic basis of epilepsy has not translated into therapeutic options able to overcome the concerning drug resistance in epilepsy, the use of these animal models remains of paramount importance [137].

Overall, the best choice of NP will depend on the drug (physicochemical attributes and potency), on what is the problem to be solved with the use of that NP, as well as on the intended route of administration. For example, i.n. administration can direct, per se, a fraction of the administered dose to the brain, which is more important for drugs that do not cross/permeate well the BBB, independently of the drug carrier, but certain drug carriers can, indeed, promote drug permeation or direct transport to the brain. In fact, some excipients, such as Tween 80 (polysorbate 80), have been described to promote adsorption/binding of apolipoprotein E to the surface of certain polymer and lipid NPs, thus, promoting active transport to the brain at the BBB [138]. Indeed, as discussed in the cited paper, important surface modification of the NPs can occur in vivo, which will dictate their fate. Drug release from the same nanocarrier in the blood circulation will strongly depend on the physicochemical attributes of the drug and its interaction with the carrier, as well as the composition, size distribution, etc., of the carrier itself. Drug loading tends to be a limiting factor in many nanosystems, but that may be less important if the drug is very potent and a smaller dose is required. Price of ingredients, manufacturing process, scalability, and robustness of reproducibility are other features that researchers often do not value, but that the industry takes into great consideration.

The biodistribution profile of the NPs within the brain is another question scarcely explored by the authors of the papers included in this review. Mainly in the case of NPs produced for diagnosis or theragnostics, we have information about the localization of these NPs in specific regions of the brain through MRI. As described in Section 5 of the manuscript (Nanoparticles for diagnosis and theragnostics), especially magnetic NPs (e.g., those composed of iron oxide) accumulate in epileptic regions of the brain, namely the hippocampus and adjacent parahippocampal cortical areas. Among these experiments, we highlighted those carried out by Fu et al. [124], proposing the use of a drug candidate with potential to interfere in epileptogenesis. Currently, there is an intense research effort focused on understanding the scientific basis of epileptogenesis and to find drugs that act on it [139], which can bring new hope for epileptic patients, namely those that are drug resistant.

Another important issue is the fact that most of the produced and tested NPs usually just help ASDs to enter the brain, increasing their concentration. This can lead to reductions in the amount of drug administration, important for patient compliance. However, it is important to remember that the formulation is more complex and more expensive than the corresponding free drug and that the cost/benefit ratio is probably not enough to attract investment from pharmaceutical companies. Indeed, considering the problems proposed in the review to be solved with the use of NPs, a reduction in drug resistance seems to be the only one that provides relevant added value regarding the currently available drugs on the market for epilepsy.

In this context, some nanoformulated drugs were already approved for other neurologic conditions. Copaxone^®^ (glatimer acetate) is an example used for multiple sclerosis, which is formulated in polymeric NPs in order to control drug clearance. In addition, morphine sulphate and cytarabine were loaded in liposomes for postoperative analgesia and for the treatment of lymphomatous meningitis, respectively. The muscle relaxant tizanidine hydrochloride was also formulated in nanocrystal NPs aiming to increase the bioavailability of the drug [68]. However, to the best of our knowledge, a drug/drug candidate formulated in a nanosystem to be used for the treatment of epilepsy is not currently being evaluated in clinical studies. This demonstrates that there is a long way to go, considering the use of NPs for the treatment of this disease.

## 7. Conclusions

Epilepsy is a complex and incapacitating neurological disease, affecting millions of people worldwide. Particularly, in the case of uncontrolled epileptic seizures, the patient’s quality of life is highly compromised. A range of structurally diverse drugs is currently being used for epilepsy management, acting through different molecular mechanisms of action. Nevertheless, despite the technological progress and the availability of many ASDs in clinical use, only 60–70% of patients with epilepsy remain seizure free when properly treated with current drugs. Therefore, the development of safer and more effective therapeutic strategies is required to fulfil an unmet medical need in this therapeutic area.

In the last few years, nanomedicines have emerged as a promising strategy to deliver therapeutic drugs towards the CNS disorders, such as epilepsy. Indeed, this approach has been used, for example, to improve the bioavailability of drugs, to enhance their solubility, to reduce toxicity, and to protect them from degradation. The claimed advantages attributed to nanosystems lead us to believe that all these limitations that are found in the current ASDs can be circumvented by their formulation in a suitable nanocarrier. Thus, the antiseizure effect of a drug can be potentiated using nanocarriers and, with brain-targeted drug delivery, the side effects can also be minimized.

Therefore, answering the question presented above, “Is nanotechnology the solution?”, we believe that the scientific community is taking the right steps, including nanocarriers as effective drug delivery systems. However, some issues still need to be clarified and deeply investigated, namely toxicological studies of brain-targeted NPs, determination of the fate of nanocarriers inside the body (e.g., biodistribution studies), and their systemic toxicity, biocompatibility, and excretion, since epilepsy is a chronic disease and long-term use is desired.

## Figures and Tables

**Table 1:** Nanoformulated antiseizure drugs, their pharmacodynamic effects, and advantages of nanoformulation.

Entry	Bioactive Compound	Main NP Composition (Size [nm], PdI)	Type of Study: Animal Model; Protocol (Positive Control)	PD Effect	Advantages of NPs	Reference
1	Lamotrigine	PLCL:Poloxamer 407 * (125, 0.184)	PK: Wistar Albino rats; i.v. single dose (oral lamotrigine tablet)	_	Increased brain penetration	[84]
2	Phenytoin	ANG-DMAEMA: NaSS:ST:ACLT-PEG-NHS:MBA (130.8 ± 22.4)	PD: Sprague-Dawley rats, amygdala kindling (chronic model); i.p., 10, 20 and 50 mg/kg, single dose (free phenytoin)	NPs lowered seizure stages and the severity of the seizure behaviour, in comparison with free phenytoin	Increased brain penetration	[85]
3	Gabapentin	Albumin:NaCl solution:Glutaraldehyde solution (141.9)	PD: Wistar rats, MES, PTZ (acute models); i.p., 50 mg/kg, single dose (free gabapentin)	Reduced the duration and average time of all phases of convulsion by polysorbate 80 coated NPs, compared with other formulations	Increased brain penetration	[86]
4	CBZ	GMS:poloxamer 188 (45.1 ± 6.7, 0.277 ± 0.03)	PD: Albino mice, PTZ, PTZ-induced kindling (chronic model); p.o., 50 mg/kg, single dose (free CBZ)	Prolonged time to death after a lethal dose of PTZ (3720 ± 245 s) compared with the free drug (2340 ± 141 s); reduced seizure score (15 and 35%, respectively)	Improved absorption profile and penetration	[87]
5	Stiripentol	Nanosuspensions stabilized with denatured soybean protein isolate	PK: Sprague-Dawley rats; p.o., single dose	_	Increased bioavailability; penetration across the intestinal barrier (in vitro and ex vivo)	[88]
6	Lacosamide	Gold-NPs:glucose (1.1, 0.252)	PD: Wistar rats, KA (chronic model); i.v., 62.5 μg/mL, single dose	Decreased amplitude and frequency of EEG-waves in both ictal and interictal stages Decreased number of seizures (not statistically significant)	Increased brain penetration	[89]
7	CBZ	PAMAM dendrimers DG4.5	Zebrafish larvae; 0.3–30 μM (free CBZ)	_	Reduced side effects Increased water solubility	[90]
8	Phenytoin	Poloxamer 235 *-PBCA (268.0 ± 2.5, 0.09 ± 0.01)	PD: Sprague-Dawley rats, lithium-pilocarpine, phenytoin-resistance (chronic models); i.p., 75 mg/kg, followed by twice daily 50 mg/kg (free phenytoin, free phenytoin + tariquidar)	Reduced seizure frequency, similar to phenytoin + tariquidar group	Reduced drug resistance Increased brain penetration	[91]
9	Phenytoin	Iron oxide NPs:silica (24.3 ± 9.93)	PD: Wistar rats, 3-MPA resistant model (P-gp overexpression) (chronic model); i.p., 120 mg/kg (free phenytoin, 75 mg/kg)	Significant reduced prevalence of clonic (40%) and tonic–clonic seizures (20%) No observed significant changes in myoclonic seizures	Reduced drug resistance	[92]
10	CBZ	Poloxamer 188:PLGA:PVA (130–150, ~0.2)	PD: Wistar rats, INH (acute model); i.v., 0.7–5 mg/kg, single dose (free CBZ)	Minimum effective dose of 1 mg/kg vs. 30 mg/kg of free compound Delayed seizure onset and reduced their duration and intensity	Reduced drug resistance	[93]
11	Lamotrigine	Poloxamer 403 and 407 *-CDI:tryptophan derivative (20)	PK: Sprague-Dawley rats, pilocarpine; i.v., 10 mg/kg, single dose (free lamotrigine)	_	Reduced drug resistance (in vitro and ex vivo) Increased brain penetration	[94]

Abbreviations: 3-MPA: 3-mercaptopropionic acid; ACLT-PEG-NHS: Acrylate-poly(ethylene glycol)-*N*-hydroxysuccinimidylester; ANG: angiopep-2; CDI: *N*,*N*′-Carbonyldiimidazole; CBZ: Carbamazepine; CHOL: Cholesterol; DMAEMA: 2-dimethylamino ethyl methacrylate; EEG: electroencephalography; GMS: glyceryl monostearate; INH: isoniazid; i.p.: intraperitoneal; i.v.: intravenous; KA: kainic acid; MBA: *N*,*N*′-methylene bisacrylamide; MES: maximal electroshock; NaSS: sodium 4-vinylbenzene sulfonate; NPs: nanoparticles; PBCA: poly(-butylcyanoacrylate; PD: Pharmacodynamic; PdI: polydispersity index; PK: Pharmacokinetic; PL: phospholipon; PLCL: poly-ε-(d,l-lactide-co-caprolactone); PLGA: Poly (lactic-co-glycolic acid); p.o.: per oral; PTZ: pentylenetetrazole; PVA: polyvinyl alcohol; SA: stearic acid; ST: styrene. * The correspondence between Pluronic brand names used in different articles and poloxamer varieties used in this table was made by consulting a review by Russo and Villa [95].

**Table 2:** Nanoformulated antiseizure drug candidates (non-approved drugs for the treatment of epilepsy), their pharmacodynamic effects, and advantages of nanoformulation.

Entry	Bioactive Compound	Main NP Composition (Size [nm], PdI)	Type of Study: Animal Model; Protocol (Positive Control)	PD Effect	Advantages of NPs	Reference
1	EGCG	PLGA:PEG, Tween 80 (168.5 ± 9.9, <0.1)	PD: C57BL/6J mice, KA (acute model); i.p., 30 mg/kg, single dose (free EGCG)	Significant reduction of the temporal lobe epilepsy patterns (56.1% versus 36.6% for free compound)	Prolonged duration of action Protection from degradation	[98]
2	Piperine	Eudragit S100: poloxamer 188 (130.2 ± 1.6, 0.195 ± 0.002)	PD: Kunming mice, PTZ (acute model); p.o., 7.5 and 15 mg/kg, single dose (free piperine)	No seizure (15 mg/kg) or reduced seizure frequency and delayed onset of seizure (7.5 mg/kg) was found for piperine nanosuspensions. Free drug failed to prevent the PTZ-induced seizure	Increased oral bioavailability Increased brain penetration	[99]
3	Curcumin	SA:lecithin (117.9)	PD: C57BL/6 mice, KA (chronic model); single dose (free curcumin)	Mice showed greater exploring ability than free curcumin in the open field test	Increased brain penetration	[100]
4	GABA	NMBAc-DMAc-PEG-2000 (124.4 ± 0.8, 0.238 ± 0.016)	PD: Wistar rats, PTZ (acute model); i.p., 100 mg/kg, single dose (free GABA)	Retarded latency time; decreased ending time and duration of seizure, compared to free GABA	Increased brain penetration	[101]
5	Curcumin	CH-ALG-STPP (50)	PD: NMRI mice, PTZ-induced kindling (chronic model); i.p., 12.5 and 25 mg/kg, daily, 10 days (free curcumin)	Decreased seizures stage and reduced duration of generalized tonic-clonic seizures, compared to vehicle and free curcumin groups	Increased aqueous solubility	[102]
6	Muscimol	DPPC-albumin-lactose (400–500)	PD: Sprague–Dawley rats, pilocarpine (chronic model); intrahippocampal injections, 5 µg (free muscimol)	The rise of the trajectory in behaviour scores slower than the positive control	Reduced side effects	[103]

Abbreviations: ALG: alginate; CH: Chitosan; DMAc: *N*,*N*-dimethyl acrylamide; DPPC: Dipalmitoylphosphatidyl-choline; EGCG: Epigallocatechin-3-gallate; GABA: *gamma*-aminobutyric acid; i.p.: intraperitoneal; KA: kainic acid; NMBAc: *N*,*N*-methylenebisacrylamide; NPs: nanoparticles; OA: oleic acid; PD: Pharmacodynamic; PdI: polydispersity index; PEG: polyethylene glycol; PLGA: Poly (lactic-co-glycolic acid); p.o.: per oral; PTZ: pentylenetetrazole; SA: stearic acid; STPP: sodium tripolyphosphate.

**Table 3:** Nanoformulated antiseizure drugs and drug candidates (not approved for epilepsy) administered by intranasal route, their pharmacodynamic effects, and advantages of nanoformulation.

Entry	Bioactive Compound	Main NP Composition (Size [nm], PdI)	Type of Study: Animal Model; Protocol (Positive Control)	PD Effect	Advantages of NPs	Reference
1	Oxcarbazepine	PLGA (256.2 ± 2.9, 0.144 ± 0.02)	PD: Wistar rats, PTZ (acute model); i.n.; 0.5 mg/kg, 3, 11 and 16 administrations (free oxcarbazepine)	Reduced symptoms and their duration	Prolonged duration of action	[111]
2	CBZ	Carboxymethyl CH (218.8 ± 2.4 nm)	PK: C57BL mice; i.n., single dose (free CBZ)	_	Increased brain penetration	[112]
3	Clonazepam	GMS:SA:compritol:OA:GO (210.2 ± 12.7, 0.197 ± 0.08)	PD: Swiss Albino mice, PTZ (acute model); i.n., 0.2 mg/kg, single dose	Prolonged convulsion onset time (64.9 s vs. 41.7 s for control) and the onset time of death (552 s versus 113.5 s for control)	Long retention at application site	[113]
4	Combination of phenytoin and fosphenytoin	Capryol 90:Im-witor 988:Kolliphor EL:Albumin	PK: CD-1 mice; i.n., 5.8 mg/kg of phenytoin equivalents, single dose (free fosphenytoin i.v. i.n. fosphenytoin solution and i.n. nanoformulation fosphenytoin only)	_	Increased brain bioavailability	[114]
5	Amiloride	OA:Tween-80:Carbitol (89.36 ± 6.19, 0.231 ± 0.018)	PD: Swiss Albino mice, ICES, PTZ (acute models); i.n., 0.5 mg/kg, single dose (free amiloride)	Higher protection than free drug; reduced onset of myoclonic jerks with clonic generalized seizures than free drug	Increased brain penetration	[115]
6	Catechin hydrate	CH:PLGA:PVA (93.46 ± 3.94, 0.106 ± 0.01)	PD: Albino rats, PTZ, ICES (acute models); i.n. 10 mg/kg, single dose (free catechin hydrate)	Higher protection in both models, compared to free compound	Increased brain penetration	[116]
7	Thymoquinone	PLGA:PVA (97.36 ± 2.01, 0.263 ± 0.004)	PD: Albino rats, ICES (acute model); i.n., 10 mg/kg, single dose (free thymoquinone)	Increased ICES threshold and decreased the recovery period, when compared to free compound	Increased brain bioavailability	[117]

Abbreviations: CBZ: Carbamazepine; CH: Chitosan; GMS: glyceryl monostearate; GO: glycerol oleate; ICES: increasing current electroshock; i.n.: intranasal; NPs: nanoparticles; OA: oleic acid; PD: Pharmacodynamic; PdI: polydispersity index; PK: Pharmacokinetic; PLGA: Poly (lactic-co-glycolic acid); PTZ: pentylenetetrazole; PVA: polyvinyl alcohol; SA: stearic acid.

## Data Availability

Data presented in this study are available on request from the corresponding authors.
